# A Voice-Activated Device Exercise and Social Engagement Program for Older Adult–Care Partner Dyads: Pilot Clinical Trial and Focus Group Study Evaluating the Feasibility, Use, and Estimated Functional Impact of EngAGE

**DOI:** 10.2196/56502

**Published:** 2024-09-12

**Authors:** Megan Huisingh-Scheetz, Roscoe F Nicholson III, Saira Shervani, Chelsea Smith, Margaret Danilovich, Laura Finch, Yadira Montoya, Louise C Hawkley

**Affiliations:** 1 Department of Medicine University of Chicago Chicago, IL United States; 2 Center for Jewish Elderly Chicago, IL United States; 3 Department of Physical Therapy Northwestern University Evanston, IL United States; 4 NORC at the University of Chicago Chicago, IL United States

**Keywords:** voice-activated device, voice-activated devices, frailty, frail, weak, weakness, technology, activity, physical activity, exercise, exercising, caregiver, caregivers, caregiving, caretaker, caretakers, caretaking, care-giver, care-givers, care-giving, care-taker, care-takers, care-taking, gerontology, geriatric, geriatrics, older adult, older adults, elder, elderly, older person, older people, ageing, aging, voice activation, digital health, technology, exercises, online exercises, participatory design, new devices, health devices, technology development, mobile phone

## Abstract

**Background:**

Maintaining exercise is essential for healthy aging but difficult to sustain. EngAGE is a socially motivated exercise program delivered over a voice-activated device that targets older adult–care partner dyads.

**Objective:**

This 10-week pilot study aimed to assess EngAGE feasibility and use, obtain user experience feedback, and estimate potential impact on function.

**Methods:**

In total, 10 older adults aged ≥65 years were recruited from an independent living residence together with their self-identified care partners. EngAGE delivered National Institute on Aging Go4Life exercises to older adults daily, while care partners received progress reports and prompts to send encouraging messages that were read aloud by the device to the older adult. Older adults’ use was tracked, and physical function was assessed at baseline and follow-up. Follow-up focus group data provided qualitative feedback.

**Results:**

On average, participants completed 393.7 individual exercises over the 10-week intervention period or 39.4 exercises/wk (range 48-492, median 431, IQR 384-481, SD 112.4) without injury and used EngAGE on an average of 41 of 70 days or 4.1 d/wk (range 7-66, median 51, IQR 23-56, and SD 21.2 days). Mean grip strength increased nonsignificantly by 1.3 kg (preintervention mean 26.3 kg, SD 11.0; postintervention mean 27.6 kg, SD 11.6; *P*=.34), and 4 of 10 participants improved by a minimal clinically important difference (MCID) of 2.5 kg. Further, the time for 5-repeated chair stands significantly reduced by 2.3 seconds (preintervention mean 12, SD 3.6 s; postintervention mean 9.7, SD 2.7 s; *P*=.02), and 3 of 9 participants improved by an MCID of –2.3 seconds. Furthermore, 3-meter usual walk performance was brisk at baseline (mean 2.1, SD 0.4 s) and decreased by 0.1 seconds (postintervention 2, SD 0.4 s; *P*=.13), although 5 of 9 participants improved by a MCID of 0.05 m/s. Qualitative results showed perceived benefits, favored program features, and areas for improvement.

**Conclusions:**

We present a pilot study of a new voice-activated device application customized to older adult users that may serve as a guide to other technology development for older adults. Our pilot study served to further refine the application and to inform a larger trial testing EngAGE’s impact on functional outcomes, a necessary step for developing evidence-based technology tools.

## Introduction

Voice-activated devices offer the possibility of assisting the rising number of older adults with maintaining physical and cognitive function, enhancing social connectivity, and accessing health and social resources from their homes [1,2]. Voice-activated devices reduce technology interface barriers by allowing users to simply talk to interact with a device [3,4]. They have shown promising early acceptability, use, and adoption among older adults, including low-income older adults [5-7]. Given the ease of use and early acceptability, commercial health care platforms incorporating a voice-activated element are on the rise [8,9]. However, the development of evidence-based health content tailored to older adults for use with voice-activated devices is in its infancy. As a result, no examples of developing voice-activated device programs for older adult users are in the literature to serve as a participatory design template.

EngAGE is a novel exercise program customized for older adults that is delivered over an Amazon Echo Show voice-activated device. The program has dual and interconnected aims to improve the physical functioning and social engagement of older adults. EngAGE supports physical functioning by delivering daily exercise routines from the National Institute on Aging (NIA) Go4Life program [10] to older adults in their home. EngAGE supports social engagement by leveraging meaningful social relationships to provide active social reinforcement that encourages behavior change [11], to provide occasions for additional social contact, and to provide passive safety oversight. EngAGE uniquely targets older adult–care partner dyads as paired users, providing a complementary resource that supports both roles. EngAGE was co-designed with older adults and their care partners through iterative, participatory design to ensure ease of adoption and meaningful content—a strategy recommended by experts in the field [12,13]. First, the concept was informally discussed with >40 stakeholders in the field through 1:1 conversations for feedback. Second, static wireframes representing possible program features were presented to predominantly minority older adults and care partners residing in the community around the University of Chicago for feedback in co-design focus groups or 1:1 interviews for homebound participants. Finally, a prototype was presented to predominantly minority older adults and care partners residing in the community around the University of Chicago for feedback in focus groups. At the final set of co-design focus groups, participants were asked to interact with the prototype as well as use a draft command tip sheet. At each stage, feedback was incorporated into the program.

As an extension of our participatory design process, we conducted a pilot study to (1) determine the feasibility of in-home administration of the EngAGE program to older adult–care partner dyads; (2) quantify the use of EngAGE during a 10-week intervention phase; (3) obtain qualitative feedback on the perceived program benefits, favored program features, and areas for improvement; and (4) estimate the potential impact of the EngAGE program on functional outcomes. These findings have relevance to clinicians and researchers exploring the utility of voice-activated devices to deliver health care resources to older adults, and to technology developers seeking to contribute useful and usable voice-activated device tools for the delivery of older adult health care resources. In an era of increasing reliance on telehealth and remote health care delivery and a shortage of geriatrics-trained health care workers [14], we anticipate a growing need for easy-to-use digital interventions for physical, social, and all other aspects of health for older adults and their care partners. Voice-activated devices are candidate technology vehicles for delivering health care programming that may be particularly suited for older adult users; however, deploying participatory design and collecting user input in feasibility studies such as this one help ensure that interventions align with older adults’ own preferences, lifestyle, and priorities to support adoption.

## Methods

### EngAGE

EngAGE is a program that delivers socially motivated exercise routines tailored to older adults in their home on voice-activated devices. The corresponding application was optimized for an Amazon Echo Show or Amazon Fire tablet but can be used on any Amazon Alexa device. It is currently not adapted for use on any other platform (eg, Google Nest). We contracted Orbita, Inc, which is a preferred Amazon Alexa programmer company, to program our EngAGE application. EngAGE leverages a software platform created by our programming partner [15] that has three user portals: (1) a browser, (2) a mobile app, and (3) a voice-activated device. Older adult users primarily interface with the voice-activated device, while care partners interface entirely with the browser and mobile application.

Older adult users activate EngAGE using voice on their screened Alexa device (eg, “Alexa, start EngAGE”). Once started, EngAGE then reads aloud any new messages from their care partner (eg, “You have a new message from [NAME]. Great job doing your exercises! Can’t wait to see you this weekend!”). Following this communication, EngAGE delivers exercise routines that alternate daily. The exercises were selected from the NIA Go4Life program [10] and were designed to be carried out with equipment found in the home (Table S1 in Multimedia Appendix 1). The subset of exercises was selected in consultation with a physical therapist who specializes in aging; they target critical, major muscle groups needed for daily functioning. For each exercise, EngAGE provides audio and visual instructions (eg, “Let’s start arm curls. Find some hand weights, water bottles or soup cans. Stand up. Hold the weights straight down at your sides, palms facing forward. Slowly bend your elbows and lift the weights toward your chest. Keep your elbows at your sides. Hold this position for 1 second. Slowly lower your arms. Do ten arm curls.”) and displays an image of a person completing the exercise. Each exercise is then accompanied by rhythmic music allowing the older adult time to perform the exercise on their own. A total of 13 strength, flexibility, and balance exercises are divided into 2 routines of 6 and 7 exercises each. Each routine begins with a 3-minute warm-up of walking around one’s home or in place. Alternating routines target different muscles to avoid overuse if completed daily. All older adult users are started at a very low intensity with the lowest number of repetitions for each exercise. Users rate the exercise difficulty after completion of each unique exercise. EngAGE then auto-adjusts the number of repetitions for that exercise in subsequent sessions. For example, if an exercise was rated “too easy” 3 times in a row, the number of repetitions would subsequently increase. On the other hand, if an exercise was rated “too difficult” one time, the number of repetitions would subsequently decrease. This process both enables gentle increases in difficulty to promote muscle building and protects against injury that could come from too rapid of a progression.

Care partners interface with the website or mobile app to view their paired older adult’s daily recommended exercise routines and to monitor exercise completion. They also receive a daily email with a summary of exercises completed, whether any exercise was rated “too difficult” by the older adult (as a safety feature), and a prompt to send an encouraging message via the website or mobile application to be read by the older adult’s voice-activated device.

### Study Design

We conducted a 12-week pilot study between May 13, 2019, and August 19, 2019, to test the feasibility and to estimate the use and potential functional impact of EngAGE among older adult–care partner dyads. The results from this pilot study were used to inform a randomized clinical controlled trial. Baseline survey and physical performance measures were assessed in person and in the homes of older adult participants. The 12-week pilot was divided into 2 phases: a 2-week run-in phase and a 10-week intervention phase. In-home setup of preprogrammed Echo Show devices and Alexa Fire tablets (eg, using anonymous, study email, Amazon accounts, and study phone numbers) was then conducted over 2 weeks. Research staff ensured connectivity, demonstrated how to use the program, and addressed privacy concerns, including demonstrating Alexa’s muting function, which is useful for protecting private conversations. During the 2-week run-in phase, participants reported any connectivity or program glitches encountered while familiarizing themselves with EngAGE. All problems were addressed and resolved before participants were asked to use the EngAGE program ad-lib over a 10-week intervention phase. Follow-up data collection, including physical performance measures and focus groups, occurred at the end of the intervention phase in the facility. Of note, 1 participant was wheelchair-bound with limited leg function, requiring lower extremity exercise adaptations that were provided in a paper supplement that was given to the participant during setup.

### Study Sample

#### Recruitment and Eligibility

After obtaining institutional review board approval, older adults and care partners were recruited together.

#### Older Adult Participants

Older adult participant recruitment activities occurred between December 10, 2018, and May 7, 2019, at a single independent living facility in Chicago, IL: a facility already equipped with Alexa Dot devices in about 150 residential apartments. Alexa Dots are voice-activated devices without a screen. The residents who participated in this study were familiar with using the Alexa Dots. The EngAGE program was optimized for use on the Echo Show device, a screened voice-activated device. We selected this participant group because it helped us isolate this study of the EngAGE program experience from the more general experience using a voice-activated device. Adults 65 years and older with unlimited Wi-Fi and data plans were eligible to participate. Older adults were excluded only if they had known moderate to severe dementia or were unable to understand the consent form in a teach-back approach. Participants with early cognitive impairment who were still able to consent were allowed to participate. Functionally impaired adults were encouraged to apply, and the use of walking devices or wheelchairs did not preclude participation. Recruitment of participants took place via in-person presentations and fliers at the independent living facility. Study participants meeting eligibility criteria were consented in person.

#### Care Partner Participants

Older adults who consented to participate were asked to identify a trusted social contact to act as their care partner. Care partners were eligible if they were 18 years or older, had unlimited Wi-Fi and data plans, and reported being comfortable using web browser and mobile applications. If older adults did not have a care partner in mind (n=2), the team worked with them to identify a staff member they knew and were comfortable with.

### Older Adult Measures

#### Demographics

Older adults self-reported their date of birth, race (Asian, Black or African-American, White, or other race), Hispanic ethnicity (yes or no), gender (female or male), education (≤high school or >high school), marital status (single, engaged or living with a partner, married or civil union, separated, divorced, widowed, or other), number of household members, and monthly household income (≤US $2000 or >US $2000 per month). They also separately reported their access to and use of the internet (yes or no) and any devices (check all that apply: computer, cell phone, smartphone, tablet, television, landline, or other).

#### Physical Function Measures

##### Physical Function

We administered 2 functional assessments at baseline and follow-up.

##### Adapted Physical Frailty Phenotype

The adapted frailty phenotype included 5 components: unintentional weight loss in the prior year (5% or 10 pounds), weakness (average of 3 dominant grip strength measures), exhaustion (2 self-reported questions from the Center for Epidemiologic Studies Depression Scale [16,17]), slowness (average of 3 15-foot usual walks), and low physical activity level (6-item version of the Minnesota Leisure Time Physical Activities Questionnaire [16,18]; Text S1 in Multimedia Appendix 2 [16-19]).

##### Short Physical Performance Battery

The SPPB consisted of three assessments: 3 static balance poses (side-by-side, semitandem, and tandem stance), a 3-meter usual walk, and 5-repeated chair stands [19] (Text S1 in Multimedia Appendix 2 [16-19]).

#### Program Use

The software platform [15] that hosts the EngAGE program recorded every exercise completed by the participant and the corresponding level of difficulty ratings throughout the intervention and stored use data on a Health Insurance Portability and Accountability Act–compliant server. Once the participant rated the level of difficulty following each exercise, the exercise was deemed “completed” regardless of the number of repetitions.

### Care Partner Measure

Care partners were asked to report their relationship to the older adult participant (eg, friend, family, spouse, or staff).

### Follow-Up Focus Groups

Three 1.5-hour focus groups of 2-4 older adult participants were held after the completion of the 10-week intervention phase. The small focus group size accommodated participant availability. This study team’s qualitative specialist (RFN) acted as the moderator for each focus group and guided the discussion using the same semistructured interview guide for each focus group to obtain feedback, with other research team members also contributing to focus group discussion. Prompts included the following topics: how EngAGE fit with pre-existing exercise habits; the role of EngAGE’s social component; participants’ current technology usage; perceptions of EngAGE’s benefits; user interface feedback; and user experience, including favored program features and areas for improvement, barriers to use, and feature evaluation. Care partners completed exploratory interviews or focus groups only, and the findings are not reported.

### Analysis

#### Analytic Approach

Data collected from the mixed methods were analyzed using several steps.

#### Older Adult Sample Characteristics

Older adult and care partner demographic characteristics were summarized for each group using the number of participants and percent of the sample for demographic categories.

#### Implementation Experiences

The total number and type of technology glitches reported during the 2-week run-in period were reported.

#### Program Use

Program use was quantified by summing the number of exercises each older adult completed over the 10-week intervention phase, and then averaging across all participants.

#### Physical Function Performance

Older adults’ physical performance measures were summarized as means (continuous measures) or frequencies (categorical measures) plus SEs and SDs for each of the 5 physical frailty phenotypes and 3 SPPB components as well as the total scale scores at baseline and follow-up. Each continuous outcome variable was assessed for normality using the Shapiro-Wilk test. Normally distributed baseline and follow-up continuous measures (average 15-foot usual walk, self-reported physical activity energy expenditure, frailty phenotype score, the fastest of two 3-meter usual walks, 5-repeated chair stands, and total SBBP score) were compared using unadjusted, paired, 2-tailed *t* tests. Nonnormally distributed baseline and follow-up continuous measures (average dominant grip strength) were compared using an unadjusted Wilcoxon matched-pairs sign rank test. Categorical variables demonstrated no change; therefore, no statistical tests were conducted. Statistical significance was set at *P*<.05. We additionally calculated the effect size of the change between pre- and postmeasurements. For each measure, we also identified the minimal clinically important differences (MCIDs) based on the literature (as available) to assess the number of participants demonstrating clinical improvement. In many cases, the MCIDs reported in the literature were not well established; therefore, we chose informed but conservative cut points. We reported the number of participants meeting the following MCID criteria: 2.5-kg increase in grip strength [20], 1-point decrease in frailty [21], 0.05-mps increase in 15-foot or 3-meter usual walk [22], 2.3-second decrease in 5 repeated chair stands [23], and 0.5-point increase in SPPB score [24]. The MCID is not established for exhaustion; self-reported physical activity on the 6-item Minnesota Leisure Time Physical Activity Questionnaire; and side-by-side, semitandem, and tandem balance performances. For these measures, we chose to report the number of participants meeting the following: (1) no longer meeting physical frailty “low physical activity” criteria (physical activity), (2) no longer meeting physical frailty “exhaustion” criteria (exhaustion), (3) number able to hold for 10 seconds pre- and postintervention (side-by-side stance), (4) number able to hold for 10 seconds pre- and postintervention (semitandem stance), and (5) number able to hold for 10 seconds pre- and postintervention (tandem stance). We did not report a clinically meaningful change in weight.

#### Qualitative Results for Perceived Benefits, Favored Program Features, and Areas for Improvement

The qualitative data were analyzed to determine perceived benefits, favored program features, and areas for improvement. Audio recordings of the focus groups were transcribed and deidentified. They were analyzed using Dedoose software [25]. Further, 2 team members (RFN and CS) independently read the transcripts to identify preliminary codes and major theme categories for qualitative analysis. Deductive themes were related to a priori topics of interest that were integrated into the focus group prompts, and inductive themes were based on topics or insights drawn from transcripts themselves. Themes and codes were then organized and compiled into an initial codebook created by the team’s qualitative specialist (RFN). The codebook was reviewed by the final coding team (RFN and MHS). Adjustments were made to the codes, themes, and definitions based on discussion options to calibrate the understanding of codes and ensure intercoder agreement of all code definitions. Then, 2 members of the team (RFN and MHS) independently reviewed the transcripts again, labeling appropriate excerpts with corresponding codes from the final codebook. Memos attached to ambiguous excerpts were discussed to reach a consensus, and where needed, adjustments to the codebook were made or recoding was accomplished. Upon completion of coding, any coding discrepancies between coders were discussed and resolved.

### Ethical Considerations

This study was approved by the University of Chicago Institutional Review Board (IRB #19-0130).

## Results

### Older Adult Sample Characteristics

The older adults represented a broad range of ages (range 65-84 years) with 7 of the 10 being 75 years or older. A majority of the sample was female (7 of 10 participants), White (9 of 10 participants), college educated (10 of 10 participants), and lived alone (8 of 10 participants). Further, 9 of 10 participants reported having access to and using a computer and smartphone (Table S2 in Multimedia Appendix 3).

### Care Partner Sample Characteristics

Each older adult identified an eligible care partner; of the 10 care partners invited to participate, all consented. Immediately after consenting, 1 discontinued due to schedule conflicts. Of the 9 remaining care partners, 3 identified themselves as a “friend,” 3 as a “child,” 2 as “staff,” and 1 as a “spouse.”

### Implementation Experiences

Having the necessary Orbita, Inc, software preinstalled on the care partner tablets and in-home setup of the Alexa devices facilitated participation. The 2-week run-in phase with easy access to technology phone support enabled the identification of 32 technical issues, most identified during the first 2 weeks. The majority were for programming glitches (n=20). Other issues addressed included requests to correct spelling errors (n=1), resolve clock inconsistency (n=1), increase the font size (n=1), address poor wireless connectivity (n=1), correct errors in exercise text instructions (n=2), clarify web application capabilities (n=2), remove a floor exercise (n=1), resolve a log-in error (n=1), resolve an EngAGE program setup problem (n=1), and resend the EngAGE program invitation email (n=1).

### Program Use

User-level analytic data indicated that the older adult participants cumulatively completed an average of completed 393.7 individual exercises over the 10-week intervention period or 39.4 exercises/wk (range 48-492, median 431, IQR 384-481, SD 112.4). Since each routine included 6-7 exercises, older adult participants completed approximately 6 exercise routines per week. Participants opened EngAGE an average of 41 of 70 days or 4.1 d/wk (range 7-66, median 51, IQR 23-56, SD 21.2 days). Further, 8 of the 10 older adult participants completed at least 2 full strength, balance, and flexibility exercise routines per week on average—the minimum recommended by the American College of Sports Medicine [26]—while 2 of the 10 participants did not meet this threshold (Table 1).

**Table 1:** Cumulative 10-week use of EngAGE by study participant.

Participant	Exercises completed, n	Days program used, n
1	140	13
2	431	59
3	449	56
4	384	50
5	398	52
6	481	66
7	48	7
8	170	23
9	492	56
10	196	28

### Physical Performance

Table 2 and Table S3 in Multimedia Appendix 4 summarize changes in physical performance in specific domains and across the frailty phenotype and SPPB scales. Functional improvements were noted in hand grip strength (mean grip strength pre 26.3 and SD 11 kg, post 27.6 and SD 11.6 kg, *P*=.34, effect size=0.32, 7/10 participants improved, 4/10 participants met MCID criteria); 5-repeated chair stands performance time (excluded wheelchair-bound participant, mean 5-repeated chair stand time pre 12 and SD 3.6 s, post 9.7 and SD 2.7 s, *P*=.02, effect size=–0.93, 6/9 participants improved, 6/9 participants met MCID criteria); and in tandem balance (excludes wheelchair-bound participant, pre 5.9 s, post 6.5 s, *P*=.78, effect size=–0.10, 4 participants held stance for 10 seconds at baseline, 3 participants at follow-up), though only change in 5-repeated chair stands performance time was statistically significant in this small pilot sample. The group (excluding the wheelchair-bound participant) had an equally brisk 15-foot and 3-meter usual walk times at baseline (mean 2.1, SD 0.4 s) and follow-up (mean 2, SD 0.4 s; 15-foot walk *P*=.86, effect size=0.06; 3-meter walk *P*=.13, effect size=–0.57), but 5/9 participants had improved usual walk times in both tests, and 3 and 5 met MCID criteria in the 15-foot and 3-meter walks, respectively. Among those not wheelchair-bound (n=9), all were capable of holding the side-by-side and semitandem balance stances for the full 10 seconds at baseline and follow-up. The mean frailty score at baseline was 0.7 (SD 0.7) and 0.0 (SD 0) at follow-up (*P*=.01) with 6/10 participants showing improvement and meeting MCID criteria. The mean SPPB score at baseline was 10.2 (SD 1.6) and 10.9 (SD 0.6) at follow-up (*P*=.22) with 5/10 participants showing improvement and meeting MCID criteria.

**Table 2:** Physical function performance measures before and after 10 weeks of EngAGE use among older adults.

Functional measures		Participants, n	Before 10 weeks				After 10 weeks				*P* value^a^		Any improvement, n		Met MCID^b^ criteria, n^c^
			Pre	SE	SD	Post		SE	SD						
**Frailty phenotype**															
	Dominant handgrip strength (kg), mean	10	26.3	3.5	11	27.6		3.7	11.6	0.33		7		4	
	Average 15-foot usual pace walk (s), mean^d^	9	4.3	0.3	0.8	4.3		0.2	0.7	0.86		5		3	
	Self-reported physical activity energy expenditure (kcal/wk), mean	10	1304.3	407	1287	1687.7		615.9	1947.7	0.56		6		2	
	Exhaustion, n	10	0	0	0	0		0	0	0		0		0	
	Self-reported weight (lb), mean	10	168.4	14.3	45.1	172.5		16.4	52	0.4		NR^e^		NR	
	Frailty total score (range 0-5), mean	10	0.7	0.2	0.7	0		0	0	0.01^f^		6		6	
**SPPB^g^**															
	Usual pace 3-meter walk (s), mean^d^	9	2.1	0.1	0.4	2		0.1	0.4	0.13		5		5	
	5 repeated chair stands (s), mean^d^	9	12	1.2	3.6	9.7		0.9	2.7	0.02^f^		8		3	
	Side-by-side stance^d^ (s), mean	9	10	0	0	10		0	0	N/A^h^		—^i^		—	
	Side-by-side stance held 10 seconds^d^, n	9	9	N/A	N/A	9		N/A	N/A	N/A		N/A		N/A	
	Semitandem stance^d^ (s), mean	9	10	0	0	10		0	0	N/A		—		—	
	Semitandem stance held 10 seconds^d^, n	9	9	N/A	N/A	9		N/A	N/A	N/A		N/A		N/A	
	Tandem stance^d^ (s), mean	9	5.9	1.5	4.4	6.5		1.2	3.6	0.78		5		—	
	Tandem stance held 10 seconds^d^, n	9	4	N/A	N/A	3		N/A	N/A	N/A		N/A		N/A	
	SPPB total score (range 0-12), mean^d^	9	10.2	0.6	1.6	10.9		0.2	0.6	0.22		5		5	

^a^Unadjusted, paired, 2-tailed *t* tests were used for normally distributed continuous variables, Wilcoxon matched-pairs sign rank test was used for nonnormally distributed continuous variables.

^b^MCID: minimal clinically important difference.

^c^Grip strength change: +2.5 kg; 15-foot usual walk: +0.05 m/s; physical activity: no longer meeting physical frailty “low physical activity” criteria; exhaustion: no longer meeting physical frailty “exhaustion” criteria; weight: not reported; frailty: –1 point; 3-meter usual walk: +0.05 mps; 5 repeated chair stands: –2.3 s; side-by-side stance: number able to hold for 10 seconds; semitandem stance: number able to hold for 10 seconds; tandem stance: number able to hold for 10 seconds; Short Physical Performance Battery score: +0.5 points.

^d^Excludes the wheelchair-bound participant.

^e^NR: Not reported.

^f^Statistically significant.

^g^SPPB: Short Physical Performance Battery.

^h^N/A: not applicable.

^i^Not available.

### Qualitative Results for Perceived Benefits, Favored Program Features, and Areas for Improvement

#### Perceived Benefits and Favored Program Features

Focus group participants described several positive outcomes resulting from their use of EngAGE, including improvement in upper and lower physical strength, balance or flexibility, knowledge gained, and social benefits (including adherence, interactions with other participants, and relationships with care partners).

Many of these comments provided “real world” examples that suggested clinically relevant strength improvements. For example, a participant attributed the improvement in grip strength from EngAGE to their ability to open a pickle jar and bottle of wine that their companions were struggling with. Another participant described the benefits derived from sit-stand exercises when using low toilets without grab bars.

Overall, balance and flexibility were less frequently mentioned than strength gains in focus group discussions of program benefits. Both participants who described flexibility improvements had not been previously doing this type of exercise, with one noting that:

I am not sure I would have chosen [the hamstring stretch], if I had been asked to pick the exercises I wanted to do.

That participant also reported balance improvements, and in both cases, these exercises were among the most difficult for her at the start of the program.

The most dramatic of the physical health benefits was described by a wheelchair-bound participant who was provided with adapted EngAGE exercises to accommodate his physical limitations. In focus groups, the participant reported:

...one of the benefits to this program is that it had side benefits to me, it helped me lose weight, helped me stop eating so much, and helped me psychologically.

The participant also reported being able to sleep through the night, due in part to the elimination of nighttime muscle stiffness and spasms, which the participant attributed to the stretching exercises.

Another benefit participants noted was the knowledge gained through the program. Further, A user praised EngAGE for providing greater knowledge about how to perform certain exercises, noting that in their prior experiences with exercise class settings:

...by the time I figured how I am supposed to do it we would be moving onto the next thing sometimes. So I like that and I could, sometimes I would pause between the first rep and the second [and] think, ‘okay, now do I know what I am supposed to be doing?’ So I like being able to do that.

Another participant whose prior exercise regime focused on recumbent biking and walking praised the program for emphasizing the need for a comprehensive range of exercise, stating:

It reminded me that I needed exercising for everything.

This was echoed by another participant who praised the greater self-awareness of the need for comprehensive physical exercise that the program provided, saying:

I just realized I was getting lazy and at our age we can't afford to be lazy because I mean the muscles are going to go and they go quickly. So that's forced me to remember and to think about how I'm doing things, and I find myself taking the hard road sometimes simply because I know I can do it and I need to do it.

Lastly, participants described positive outcomes related to the social component of EngAGE for motivating adherence, contributing to insight regarding the fitness benefits, and strengthening social relationships. Multiple participants reported that having a partner who could monitor their progress aided with their adherence to the program. Further, 1 participant also described the questions that she received from her care partner as “creating a dialogue, forcing you to think about it a little bit and that does help.” These interactions with care partners were occasions for reminding participants of the progress that they had made: “It was always something that would make you, really force you to think about, geez, yes, the sit-stand is really helping. I've really improved that.”

Participants also described benefits related to their relationships with their care partners and other participants. Some of this was related to the encouragement and social support that they received. Interestingly, 2 participants felt that this sort of “rah, rah, your chief cheerleader” support was better suited to the EngAGE messaging format, noting “it might seem a little bit corny if she said it to you in-person but in the email, in the message, it seems great.” Since all participants resided in the same building, participants also described their camaraderie with each other and reported interest in program features that would permit communication across exercisers, with 1 suggesting an interface “almost like a Facebook page.” Further, 1 participant also described the benefits of EngAGE for participants who prefer to exercise alone but still desire social reinforcement, noting:

That doesn't mean I don't like to talk about it. It doesn't mean I don't want to interact with somebody about it. I just don't want anybody there while I'm doing it.

Finally, 1 participant whose care partner was an adult child described the messaging functionality as an opportunity for increased communication on topics unrelated to exercise. Another participant whose care partner was a friend noted that the program “certainly added to communication between us, although we still usually fairly often communicate.”

#### Areas for Improvement

The most common program criticism was the lack of exercise variety. Study participants wanted more than 2 alternating routines in a week and additional exercise types as skill levels increased. Multiple participants reported the 2 daily alternating routines became “boring” or “tedious.”

Additionally, some participants felt the length of exercise instructions was longer than required, especially once familiar with the exercise, with 1 older adult noting that “the full prompt every time, got to be a little bit much,” and others reporting they began the exercise while still listening to the instructions. In other cases, participants mentioned the length of the repeated exercise sets, with 1 noting:

...when I would hit the third one it was starting, I was starting to feel like, is this ever going to end?... And yeah, that was hard. And I'll be honest, I didn't always do the third set.

Other feedback centered around the functionality of the EngAGE interface. Multiple participants, particularly the more experienced exercisers, requested more control over exercise difficulty rather than the automatic changes based on the exercise rating. Other sources of user frustration included difficulty pausing and returning to the same place when unexpectedly interrupted by a phone call or visitor, difficulty successfully skipping instructions, and exercises not registering as completed. Further, 1 participant found the audio instructions more useful than the written instructions or pictures on the screen:

...I basically ignored the screen and listened to the verbal.

Another theme emerged about care partners not meeting expectations. For example, 1 participant emphasized that:

The quality of partner is an issue.

Another mentioned that:

I was kind of hoping that she would get a little more into it, I guess, than she did.

She further explained that:

I would have liked her to ask more questions about the exercises... I think you need more of a dialogue than just a pat on the back or their head.

## Discussion

### Summary of Findings

This paper summarizes a participatory design approach for developing new voice-activated device programs with older adult users, and it reports results from the final stage of participatory design, a pilot study. In this pilot study, we found the voice-activated device and EngAGE were feasible to set up on-site. The program was moderately used on average by study participants but with a wide range. User feedback provided targeted opportunities to improve the user experience. We estimated the potential impact on physical function to inform the sample size needed for a subsequent efficacy trial. We found participants were the most likely to experience improvement in chair stand time performance of all outcomes assessed. The results from this pilot study offered feasibility considerations for future aging technology studies, provided a reference range for voice-activated device program use among older users for future studies, recommended sensitive objective and patient-reported outcome targets for older adult exercise trials, and introduced a shift in targeted technology users from the older adult to the older adult–care partner dyad.

This study provided several key feasibility implications for future voice-activated device intervention studies in older adults. First, the in-home device setup and data collection provided for older adults greatly streamlined onboarding and facilitated the completion of important objective functional measures but will be challenging for large-scale studies. Potential strategies to address home technology setup needs for larger scale studies include (1) leveraging the paired care partner to assist the older adult with device setup, (2) using setup instruction manuals that have undergone participatory design with older adults to ensure ease of use paired with telephone support, and (3) partnering with an organization that has existing on-the-ground technology support teams. Remote functional data collection may be carried out using videoconferencing [27] or by leveraging wearable sensors in the future [28-32]. Second, our 2-week run-in phase helped address many technical issues before the intervention phase that could have interfered with a successful intervention adoption. Larger technology intervention studies should consider budgeting for phone and on-the-ground technology support, particularly in the first few weeks of technology use. Third, privacy concerns were not the participation barrier we anticipated they could be. We heavily addressed privacy issues during consent and provided strategies for maintaining privacy during device setup, which may have alleviated concerns up front. Everyone in this group was an existing Alexa user, so future studies may encounter these concerns more frequently. Finally, we had to adapt the EngAGE exercises to accommodate a wheelchair-bound participant; future studies should anticipate functionally limited users and prepare alternative exercises in advance.

In this study, the older adults who were familiar with Alexa at baseline used this program on more than half of the days per week on average, but the wide range of EngAGE program use was an important finding for future tech researchers. Adherence will probably be lower among users with less familiarity with Alexa at baseline or those who are more functionally impaired; therefore, a longer intervention period may be necessary to see significant functional gains in a trial targeting these subgroups. Further, 1 option to boost voice-activated device program use among older adults would be to leverage regular visits from care partners such as a state-sponsored homemaker or paid care partners. The care partners could provide direct technology support and help the older adult use the EngAGE program or other healthy aging programs in the home at each visit.

In our pilot sample of mostly current exercisers, chair stand times improved significantly, and we observed a nonsignificant improvement in dominant grip strength over the 10-week intervention phase. These findings inform the sample size needed for a larger EngAGE efficacy trial in older adults and may serve to inform similar technology-based exercise trials in older adults. The fact that a relatively high-functioning group of older adults exhibited improvement in some aspects of physical performance suggests EngAGE or similar programs may have even greater potential to improve outcomes for less robust older adults.

In addition to informing adjustments needed to the EngAGE program, the results of our qualitative analyses provided insights regarding the potential subjective outcome targets for larger technology-based exercise trials. Several participants self-reported meaningful functional gains in activities requiring the hand (opening a jar) and proximal hip (improvement in standing up from a low seat), suggesting these are sensitive patient-reported outcomes for a larger trial. A small number of participants also noted subjective improvement in flexibility, weight loss, diet, mood, and sleep, which may be helpful outcomes to include in larger studies. Finally, while nearly all older participants in the pilot study were exercisers at baseline, participants described gaining knowledge from the comprehensiveness of the exercises—we did not measure knowledge gains directly but would be important for future studies.

Older adult dyadic relationships within their social network are important to healthy aging [33], but health care technology programs infrequently target both users as a unit. The EngAGE program uniquely leverages existing social relationships to motivate activity while allowing older users to exercise alone and at their own pace—both welcomed features in this pilot sample. This strategy aims to both improve older adult physical independence and simultaneously increase opportunities for social engagement. Most older adult exercisers reported that the care partners successfully provided accountability, promoted adherence, increased social communication, and even encouraged older adults to reflect on their progress. In technology interventions, this partnership could be leveraged even more to assist with things such as device setup, addressing technical issues, monitoring safety, or even participating in a program such as EngAGE with the older user. This pilot study also showed that not all social relationships are equally effective. The most positive responses came from those who had care partners who were thoughtful, creative, and engaging. Further, one strategy for increasing the odds of receiving meaningful exercise motivation might be to increase the number of care partners paired with each older adult or to allow the older adult users to be connected. For example, children, grandchildren, and friends could be paired with a single older adult—a feature that is available on the EngAGE platform. Another strategy might be to provide care partners with tips for motivating healthy behavior. A key message from this pilot is the critical nature of care partners in implementing and sustaining healthy aging behavior among older adults.

### Study Limitations

By design, our pilot sample was small, the team and participants were unblinded and the participants’ familiarity with Alexa devices enabled us to primarily test the EngAGE program including its use, experience, and potential impact. However, these restrictions limit the generalizability of our findings and could introduce bias. Many of those recruited were higher functioning and better resourced than frail older adults (our ultimate target users). Before this feasibility study, however, our participatory design process included less resourced, physically limited, less technology savvy, and predominantly minority older adults. This sample was selected because they were existing Alexa users and could provide feedback on the EngAGE program experience and not the Alexa experience, allowing us to identify areas we could improve before testing in a more vulnerable group. Future participatory design studies must include functionally impaired and less tech-savvy older adults during development so that their needs and concerns are addressed in the program design.

### Conclusion

Voice-activated devices hold great promise for overcoming many technology use challenges for older adults, making them a potential vehicle for delivering healthy aging resources broadly to older adults [2,34]. EngAGE is unique in that it targets older adult–care partner dyads and underwent an iterative participatory design process throughout development with vulnerable users to improve the likelihood of perceived ease of use, perceived usefulness, and technology adoption among all older adults. Our pilot study demonstrated that screened voice-activated devices are well-suited to remote delivery of exercise routines that do not require specialized equipment. Using the NIA Go4Life content, EngAGE appears capable of producing statistically and clinically significant improvements in objective and subjective physical function measures. The social component of EngAGE was, overall, viewed positively as an exercise motivator and as a means of strengthening bonds and increasing communication between dyad members. This study also has important feasibility implications for larger technology program trials, including the need for 1:1 device setup infrastructure and technology support. These findings are relevant to all future aging technology studies but especially to voice-activated device studies.

## Appendix

Multimedia Appendix 1 Exercises from the National Institute on Aging's Go4Life Program included in the EngAGE Program.

Multimedia Appendix 2 Detailed physical function measure descriptions.

Multimedia Appendix 3 Older adult sample characteristics (N=10).

Multimedia Appendix 4 Physical function performance measures before and after 10 weeks of EngAGE use among older adults including effect sizes.

## Abbreviations

NIA: National Institute on Aging
SPPB: Short Physical Performance Battery

## Data Availability

This study’s data are available from the corresponding author (MHS) upon reasonable request and after completion of a Data Use Agreement and Institutional Review Board assessment.
