# Supplementary material for: A Voice-Activated Device Exercise and Social Engagement Program for Older Adult–Care Partner Dyads: Pilot Clinical Trial and Focus Group Study Evaluating the Feasibility, Use, and Estimated Functional Impact of EngAGE
Source: JMIR Aging. 2024 Sep 12;7:e56502. doi: 10.2196/56502 (PMC11427853; doi:10.2196/56502)
Supplement: Multimedia Appendix 1 [file aging_v7i1e56502_app1.docx]

**Table S1**

| **Go4Life Exercises** | **EngAGE Exercises** | **EngAGE Routine** |
| --- | --- | --- |
| **Flexibility** | | |
| Getting down on the floor |  |  |
| Getting up from the floor |  |  |
| Neck |  |  |
| Shoulder |  |  |
| Shoulder and upper arm |  |  |
| Upper body |  |  |
| Chest | Chest | Tuesday/Thursday/Saturday |
| Ankle Stretch |  |  |
| Calf Stretch | Calf Stretch | Tuesday/Thursday/Saturday |
| Back of leg stretch (floor) |  |  |
| Back of leg stretch (bench) |  |  |
| Thigh Stretch (floor) |  |  |
| Thigh stretch (standing) | Thigh stretch (standing) | Monday/Wednesday/Friday/Sunday |
| Hip |  |  |
| Back Stretch 1 |  |  |
| Back Stretch 2 |  |  |
| Upper back |  |  |
| Lower back |  |  |
| Buddy Stretch |  |  |
| **Strength** | | |
| Hand grip | Hand grip | Tuesday/Thursday/Saturday |
| Wrist curl |  |  |
| Overhead Arm Raise | Overhead Arm Raise | Monday/Wednesday/Friday/Sunday |
| Front Arm Raise |  |  |
| Side Arm Raise |  |  |
| Arm curl | Arm curl | Tuesday/Thursday/Saturday |
| Arm curl with resistance band |  |  |
| Wall push-up | Wall push-up | Monday/Wednesday/Friday/Sunday |
| Elbow extension |  |  |
| Back leg raise | Back leg raise | Monday/Wednesday/Friday/Sunday |
| Side leg raise | Side leg raise | Monday/Wednesday/Friday/Sunday |
| Chair stand (with chair) | Chair stand (with chair) | Monday/Wednesday/Friday/Sunday |
| Chair dip | Chair dip | Tuesday/Thursday/Saturday |
| Knee curl |  |  |
| Leg straightening |  |  |
| Toe stand (standing) | Toe stand (standing) | Tuesday/Thursday/Saturday |
| Lower back |  |  |
| Seated row with resistance band |  |  |
| **Balance** | | |
| Stand on one foot (with chair) | Stand on one foot (with chair) | Monday/Wednesday/Friday/Sunday |
| Heel toe walk |  |  |
| Balance Walk  Getting up |  |  |
