# Supplementary material for: A Voice-Activated Device Exercise and Social Engagement Program for Older Adult–Care Partner Dyads: Pilot Clinical Trial and Focus Group Study Evaluating the Feasibility, Use, and Estimated Functional Impact of EngAGE
Source: JMIR Aging. 2024 Sep 12;7:e56502. doi: 10.2196/56502 (PMC11427853; doi:10.2196/56502)
Supplement: Multimedia Appendix 2 [file aging_v7i1e56502_app2.docx]

**Text S1**

*Physical Function Measures.* We administered two functional assessments at baseline and follow-up.

1) *Adapted* *Physical Frailty Phenotype* [1]. The adapted frailty phenotype includes 5 components: a) Unintentional weight loss: OAs were asked to self-report any weight loss in the prior year and intentionality of any loss. If they reported unintentional weight loss of ≥10 pounds or ≥5% in prior year, they were given one point. b) Weakness: To assess weakness, dominant hand grip strength (kg) was assessed three times using a Jamar dynamometer and averaged. A point was assigned if participants were weaker than published gender- and BMI-adjusted cut-points [1]. c) Exhaustion: OAs were asked to self-report exhaustion, which was measured using two questions from the CES–D scale [1, 2]. The two items included “In the last week, how often did you feel that everything you did was an effort?” and “In the last week, how often did you feel that you could not get going?” Answer choices included “None of the time,” “Some of the time (1-2 days),” “A moderate amount of the time (3-4 dates),” or “Most of the time (> 4 days). Participants who reported “A moderate amount of time” to either question were assigned a point for exhaustion. d) Slowness: Slowness was assessed with a timed, 15-foot walk performed at a ‘usual’ pace. OAs completed the walk three times and performance times recorded to the tenth decimal were then averaged. If the average usual walking time was below gender- and height-adjusted cut-points, a point was assigned [1]. e) Low physical activity level: The 6-item Minnesota Leisure Time Physical Activities Questionnaire (6-MLTPAQ) was administered to assess the OA’s typical level of physical activity. Specifically, OAs were asked about the frequency of engaging in six different types of activities (walking, chores, gardening, general exercise, mowing the lawn, and golfing) during the prior (baseline: 12 months or follow-up: 10 weeks) [3]. Average weekly physical activity kilocalorie expenditure was then calculated.

2) *Short Physical Performance Battery (SPPB)* [4]. The SPPB consisted of three assessments: 3 static balance poses; a 3-meter usual walk; and 5-repeated chair stands [4]. Balance testing included asking OAs to hold a side-by-side, semi-tandem, and tandem stances for 10 seconds each. Each task was demonstrated by a team member prior to administration. If the participant or interviewer felt unsafe, the task was not attempted, and if an easier pose was not held for 10 seconds, the more complex pose(s) was/were not attempted. Time to the tenth decimal was recorded for each pose. Combined performance on the three stances was used to generate a score ranging from 0 to 4, with 4 indicating intact balance as previously described [4]. Participants were then asked to walk 3 meters at their ‘usual’ pace. The faster of the two performance times recorded to the tenth decimal was used to generate a score ranging from 0 to 4 as previously described, with 4 indicating a brisk walk [4]. Participants were also then asked to complete the chair stands exercise using a straight back chair without wheels. Participants were instructed to plant both feet firmly on the ground and to fold arms across the chest. A single chair stand was attempted first. If successful, participants were asked to perform five serial chair stands as quickly as possible. The time to the tenth decimal was recorded after the participant completely stood up on the fifth stand and this was used to generate a score ranging from 0-4 as previously described, with 4 indicating a brisk performance [4].

1. Fried LP, Tangen CM, Walston J, Newman AB, Hirsch C, Gottdiener J, et al. Frailty in older adults: evidence for a phenotype. J Gerontol A Biol Sci Med Sci. 2001 Mar;56(3):M146-56. PMID: 11253156.

2. Radloff LS. The CES-D Scale: A Self-Report Depression Scale for Research in the General Population. Applied Psychological Measurement. 1977;1(3):385-401. doi: 10.1177/014662167700100306.

3. Eckel SP, Bandeen-Roche K, Chaves PH, Fried LP, Louis TA. Surrogate screening models for the low physical activity criterion of frailty. Aging Clin Exp Res. 2011 Jun;23(3):209-16. PMID: 21993168. doi: 8029 [pii].

4. Guralnik JM, Simonsick EM, Ferrucci L, Glynn RJ, Berkman LF, Blazer DG, et al. A short physical performance battery assessing lower extremity function: association with self-reported disability and prediction of mortality and nursing home admission. J Gerontol. 1994 Mar;49(2):M85-94. PMID: 8126356.
