# Supplementary material for: A Voice-Activated Device Exercise and Social Engagement Program for Older Adult–Care Partner Dyads: Pilot Clinical Trial and Focus Group Study Evaluating the Feasibility, Use, and Estimated Functional Impact of EngAGE
Source: JMIR Aging. 2024 Sep 12;7:e56502. doi: 10.2196/56502 (PMC11427853; doi:10.2196/56502)
Supplement: Multimedia Appendix 3 [file aging_v7i1e56502_app3.docx]

**Table S2.**

| Older Adult Characteristics | n | % |
| --- | --- | --- |
| Age (in years) |  |  |
| 65-74 | 3 | 30 |
| 75-84 | 7 | 70 |
| Gender |  |  |
| Female | 7 | 70 |
| Male | 3 | 30 |
| Race/Ethnicity |  |  |
| White | 9 | 90 |
| Asian | 1 | 10 |
| Education |  |  |
| College graduate | 7 | 70 |
| Post graduate | 3 | 30 |
| Income (monthly) – (n=8) |  |  |
| < $3,000 | 2 | 25 |
| $3,000 - $5,999 | 2 | 25 |
| $6,000 - $8,000 | 2 | 25 |
| >$8,000 | 2 | 25 |
| Have access to technology |  |  |
| Computer | 9 | 90 |
| Smartphone | 9 | 90 |
| Internet access | 10 | 100 |
| Internet use | 10 | 100 |
| Living alone | 8 | 80 |
| Walking device use |  |  |
| Walker/cane | 1 | 10 |
| Wheelchair | 1 | 10 |
| Physical activities (in last 12 months) |  |  |
| Walked for exercise | 8 | 80 |
| Moderate/extraneous chores | 2 | 20 |
| Gardened | 2 | 20 |
| General exercise/Calisthenics | 7 | 70 |
| Played golf | 1 | 10 |
