# Supplementary material for: A Voice-Activated Device Exercise and Social Engagement Program for Older Adult–Care Partner Dyads: Pilot Clinical Trial and Focus Group Study Evaluating the Feasibility, Use, and Estimated Functional Impact of EngAGE
Source: JMIR Aging. 2024 Sep 12;7:e56502. doi: 10.2196/56502 (PMC11427853; doi:10.2196/56502)
Supplement: Multimedia Appendix 4 [file aging_v7i1e56502_app4.docx]

**Table S3.**

|  | n | Pre | SE | SD | Post | SE | SD | Test*^c^* | *P-value^c^* | Effect Size | Any Improve-ment (#) | Met MCID Criteria (#)^b^ |
| --- | --- | --- | --- | --- | --- | --- | --- | --- | --- | --- | --- | --- |
| *Functional Measures* |  |  |  |  |  |  |  |  |  |  |  |  |
| **Frailty Phenotype** |  |  |  |  |  |  |  |  |  |  |  |  |
| Dominant handgrip strength (mean, kg) | 10 | 26.3 | 3.5 | 11.0 | 27.6 | 3.7 | 11.6 | z=-0.97 | 0.33 | 0.32 | 7 | 4 |
| Average 15-foot usual pace walk (mean, seconds)^a^ | 9 | 4.3 | 0.3 | 0.8 | 4.3 | 0.2 | 0.7 | t=0.18 | 0.86 | 0.06 | 5 | 3 |
| Self-reported physical activity energy expenditure (mean, kcal/week) | 10 | 1304.3 | 407.0 | 1287.0 | 1687.7 | 615.9 | 1947.7 | t=-0.61 | 0.56 | 0.19 | 6 | 2 |
| Exhaustion (#) | 10 | 0 | -- | -- | 0 | -- |  | -- | -- | -- | -- | -- |
| Self-reported weight (mean, pounds) | 10 | 168.4 | 14.3 | 45.1 | 172.5 | 16.4 | 52.0 | t=-0.89 | 0.40 | 0.28 | -- | -- |
| Frailty total score (mean, range 0-5) | 10 | 0.7 | 0.2 | 0.7 | 0.0 | 0.0 | 0.0 | t=3.28 | **0.01** | -1.04 | 6 | 6 |
| **Short Physical Performance Battery** |  |  |  |  |  |  |  |  |  |  |  |  |
| Usual pace 3-meter walk (mean, seconds) ^a^ | 9 | 2.1 | 0.1 | 0.4 | 2.0 | 0.1 | 0.4 | t=1.71 | 0.13 | -0.57 | 5 | 5 |
| 5 Repeated chair stands (mean, seconds) ^a^ | 9 | 12.0 | 1.2 | 3.6 | 9.7 | 0.9 | 2.7 | t=2.80 | **0.02** | -0.93 | 8 | 3 |
| Side-by-side stance ^a^ (mean, seconds) | 9 | 10 | 0 | 0 | 10 | 0 | 0 | -- | -- | -- | -- | -- |
| Side-by-side stance held 10 seconds ^a^ (#) | 9 | 9 | -- | -- | 9 | -- | -- | -- | -- | -- | -- | -- |
| Semi-tandem stance ^a^ (mean, seconds) | 9 | 10 | 0 | 0 | 10 | 0 | 0 | -- | -- | -- | -- | -- |
| Semi-tandem stance held 10 seconds ^a^ (#) | 9 | 9 | -- | -- | 9 | -- | -- | -- | -- | -- | -- | -- |
| Tandem stance ^a^ (mean, seconds) | 9 | 5.9 | 1.5 | 4.4 | 6.5 | 1.2 | 3.6 | t=-0.29 | 0.78 | -0.10 | 5 | -- |
| Tandem stance held 10 seconds ^a^ (#) | 9 | 4 | -- | -- | 3 | -- | -- | -- | -- | -- | -- | -- |
| SPPB Total Score (mean, range 0-12) ^a^ | 9 | 10.2 | 0.6 | 1.6 | 10.9 | 0.2 | 0.6 | t=-1.33 | 0.22 | 0.44 | 5 | 5 |
